# Determinants of adherence to dental treatment of socially vulnerable adolescents: a cohort study

**DOI:** 10.1186/s13104-021-05525-8

**Published:** 2021-03-25

**Authors:** Jaqueline Vilela Bulgareli, Karine Laura Cortellazzi, Luciane Miranda Guerra, Gláucia Maria Bovi Ambrosano, Armando Koichiro Kaieda, Inara Pereira da Cunha, Fabiana de Lima Vazquez, Antonio Carlos Pereira

**Affiliations:** Department of Health Sciences and Children’s Dentistry, Piracicaba Dental School, State University of Campinas, Av. Limeira, 901, P.O. BOX 52, Piracicaba, São Paulo 13414-903 Brazil

**Keywords:** Treatment adherence and compliance, Adolescents, Social vulnerability

## Abstract

**Objective:**

Different studies with adolescents address the difficulty they have to adhere to oral dental treatments. Therefore, better understanding the processes involved in adherence to treatment in this population is necessary. The aim of this study was to investigate the factors that influence adherence to dental treatment in socially underprivileged adolescents in primary care.

**Results:**

Non-adherence to treatment showed high rate in the studied sample (49.5%). Family income (p = 0.039) and number of individuals in the family (p = 0.003) were associated with non-adherence to dental treatment. It is concluded that the adolescents’ social vulnerability condition resulted in situations that are incompatible with adherence, which hinders dental treatment and health service planning.

## Introduction

Adolescents constitute a population exposed to risk of developing major oral diseases, such as dental caries and periodontal disease. The World Health Organization (WHO) recently reported that most children and adolescents show signs of gingivitis [1], whose prevalence is up to 80% in adolescents [2]. According to the latest Brazilian nationwide oral health survey, more than half of the adolescents aged 15 to 19 years show signs of the disease, such as bleeding (9.7%), calculus (28.6%) and periodontal pockets (10.8%) [3].

Regardless of the disease, adolescents have greater difficulty in adhering to treatment than younger individuals [4]. Therefore, better understanding the prevalent processes involved in this population’ adherence to disease treatment is necessary.

The literature shows a great number of studies about the different concepts regarding "adherence". This can be defined as an approach to the maintenance or improvement of health in order to reduce the signs and symptoms of a disease [5], but also by the degree of compliance with therapeutic measures, whether or not with use of medication [6]. It is a complex behavioral process, heavily influenced by the environment, by health care professionals and medical care**.** When individuals fully follow the treatment they are classified as “adherent”; when they quit the treatment they are classified as “quitter” or “non-adherent”; and finally there are the “persistent,” within the “non-adherent” group, which are those individuals that attend the appointments, but do not follow the treatment [7].

Considering such factors, professionals could understand the expectations and characteristics of individuals who do not follow the recommended treatment, which enables more individualized interventions to improve adherence and hence provide a service with improved quality. In this context, longitudinal studies with adolescents contribute to the determination of individual and contextual determinants associated with adherence to dental treatment and the tracing of variables that hinder the access of socially vulnerable adolescents.

## Main text

### Methods

This is a longitudinal analytical study conducted in the city of Piracicaba, São Paulo, Brazil, between 2014 and 2015. The study was approved by the Research Ethics Committee, protocol 027/2011.

This study was directed to adolescents aged 15 to 19 years living in the area covered by 34 Family Health Units (FHU) in the city of Piracicaba. Among them, there are 12 units with oral health teams (dentist and dental assistant). These adolescents were enrolled in state schools in their territorial reference.

Family health teams provide primary care to about 1,000 families in the areas with greater social exclusion index of the municipality (≤ -0.75). The value of this index is evaluated by the Institute for Research and Planning of Piracicaba (IPPLAP, in Portuguese) and ranges from -1 (worst condition) to 1 (best condition) [8]. On average, 320 adolescents aged 15 to 19 years were enrolled in each of the 34 units, totaling approximately 11,000 individuals. Adolescents who participated in this study lived in areas with greater social exclusion, that is, they were in situation of social vulnerability.

The research included the adolescents covered by the 34 FHUs in the municipality. A sampling error of 5%, DMFT = 5.16 with SD = 4.54, sampling loss of 20% and a 95% confidence level were considered, obtaining a sample of 1,428 randomly selected individuals. Of this total, 249 did not show up on examination day. Thus, 1,179 adolescents were examined. The sample exclusion criteria were individuals with systemic diseases, communication difficulties, or neuromotor problems, severe hypoplasia and orthodontic brace. Individuals those absent on the examination day were also excluded from the sample. Adolescents served in the health care unit were considered as inclusion criteria.

About 18 months after the completion of the initial examination, the researchers conducted an active search in order to locate the adolescents who were referred to treatment in the initial phase.

Of the total 1,179 adolescents examined in the initial phase, 474 (40.2%) of them needed dental treatment and 705 received oral health promotion and prevention guidelines. Those who needed treatment were asked to schedule an appointment at the family health units, which were already prepared to receive them, for the treatment. In the units with no dentists, a referral form was given to the adolescent, who was told to go to the reference unit for treatment. The criterion for scheduling or referral was the presence of caries and/or periodontal disease.

Of the 474 participants surveyed, 325 (68.6%) were reexamined, of which 164 (50.5%) adhered to dental treatment (adherence group) and 161 (49.5%) did not adhere (non-adherence group). A total of 149 adolescents were not reexamined due to change in address and telephone number (n = 131), transfer to other municipalities (n = 9) and refusal to participate in the study (n = 9). As these adolescents were not found in the final phase of the study and did not schedule appointment at the FHU, it was not possible to know whether they underwent the treatment or not in another public service or in the private sector, so they were not included in the analysis. The final sample (325 adolescents) provided a test power of 0.80 with a 95% confidence level in the analyses performed considering adhesion of 50.0%.

The examinations were performed in the FHU, by two examiners (previously calibrated and helped by two note-takers). Data were collected with reference to the clinical characteristics: caries by the DMFT index (total decayed, missing and filled teeth) and periodontal disease (Community Periodontal Index-CPI) [9].

#### Study variables

At Level 1, we studied individual clinical sociodemographic (sex, number of people in the family), access to the service (reason to seek the dentist), reported pain (pain in the last 6 months), Oral Impacts on Daily Performances (OIDP) [11] and Family Adaptability and Cohesion Scale (FACES III questionnaire), validated in Brazil [12]. At Level 2, we analyzed the contextual variable: percentage of families in the neighborhood with income from 0.5 to 1 minimum wage. The minimum wage in Brazil at the time of data collection was US$ 1,796.70. In clinical evaluation, we considered the presence of caries, pain, abscess and/or periodontal disease, according to the WHO criteria [9].

A semi-structured questionnaire based on the Goes model [10] was applied to the set of individual variables (sex, income from 0.5 to 1 minimum wage, number of people in the family). In order to investigate the reason that led the adolescent to seek the dentist, the following question was asked: "What is the most frequent reason for you to seek the dentist?" To this end, the respondent could choose from the following answers: "for frequent checking," "only when I have a problem," and "I do not know/I do not remember."

#### Data analysis

Adherence to dental treatment was considered as response variable. Using the descriptive analysis data, multilevel logistic regression models were estimated by the GENMOD procedure, using the statistical program SAS. In the analysis, the individuals' variables were considered as level 1, neighborhood as level 2, and the setting model was evaluated by the QIC (Quasi Likelihood Under Independence Model Criterion). Initially, a model with only the intercept was estimated (model 1). Next, variables of individuals were tested (model 2). In model 3, the significant variable in model 2 and including contextual variable remained (p < 0.05).

### Results

The mean age of the adolescents reexamined was 17 years (standard deviation = 1.3). Among them, 188 (57.8%) were female and 137 (42.2%) were male. Table 1 presents the descriptive analysis of individual variables studied in the initial phase. Regarding the reason to seek dental treatment, 34.2% answered that they sought the dentist to treat the teeth, and 36.6% had experienced tooth pain in the last 6 months.

**Table 1:** Distribution and frequencies of the variables evaluated in the sample of adolescents that needed dental treatment (Initial phase) 2013–2014

Variables	n	%
Gender		
Female	188	57,8
Male	137	42,2
Reason to go to the dentist		
Pain	55	16,9
Teeth extraction	12	3,7
Dental treatment	111	34,2
For checking	54	16,6
Cleaning, fluoride	41	12,6
Other	26	8,0
Did not answer	26	8,0
Dental pain in the last 6 months		
Yes	119	36,6
No	182	56,0
Do not know/do not remember	20	6,2
Did not answer	4	1,2

Table 2 shows that the number of people in the family ranged from 1 to 6, with a median 4, and the percentage of families with income from 0.5 to 1 minimum wage ranged from 4.1 to 16.9%, with median of 10.4%.

**Table 2:** Median, minimum and maximum of individual and contextual variables evaluated in the sample (initial phase), 2013–2014

Variables	Median	Minimum–maximum
OIDP	0	0–135
Family cohesion	32	13–46
Number of people in the family	4	1–6
% income 0.5 to 1*	10.4	4.1–16.9

*OIDP* oral impact on daily performance

* % of families with income from 0.5 to 1 minimum wage (the minimum wage at the time of data collection was US$ 1.796,70)

Table 3 shows the multilevel model for the response variable adherence to dental treatment (yes and no). Model 2 showed an association between non-adherent individuals and number of individuals in the family situation (p = 0.003). Higher percentage of the non-adherence group was also observed in adolescents from neighborhoods of families with lower incomes (p = 0.039), according to the model.

**Table 3:** Multilevel model for adherence to dental treatment, 2014–2015

Variables	Model 1			Model 2			Model 3		
	Estimate	SE	p-value	Estimate	SE	p-value	Estimate	SE	p-value
Intercept	0.0062	0.1296	0.9621	1.0025	0.3361	0.0029	1.9146	0.5766	0.0009
Individual level									
Number of people in the family				− 0.2538	0.0700	0.0003	− 0.2432	0.0678	0.0003
Contextual level									
% income 0.5 to 1*							− 0.0846	0.0411	0.0396
QIC	453.2737				440.0044			437.7239	

*SE* standard error

* % of families with income from 0.5 to 1 minimum wage (the minimum wage at the time of data collection was US$ 1.796,70)

### Discussion

The results showed that socioeconomic conditions (family income and number of people in the family) were associated with non-adherence to dental treatment among socially vulnerable adolescents. This finding is important, since, in this study, access to dental treatment was guaranteed to patients; however, the behavior of adhering to the professionals’ guidelines was not adopted, and the adolescents did not return for consultation.

Thus, it is observed that, in addition to socioeconomic conditions contributing to the onset of oral problems, this is a factor that operates in a complex way on health. Economic factors lead to a lower perception of oral health needs [13] and to reduced opportunities for study, leisure, and work, which can impact subjects in decision-making aimed at protecting their own health [14].

Therefore, special attention should be directed to more socially vulnerable adolescents, considered "underprivileged," because social, political and economic inequality has a direct influence on family dynamics and, therefore, increase the personal and social risk situation experienced by these individuals. Additionally, adolescents, in their capacity as "developing individuals," have an intrinsic condition of vulnerability, lacking physical, mental and moral care, that is, a comprehensive understanding of their needs [15].

In the results of this study, individuals who did not adhere to dental treatment were those from the lower-income neighborhood families (0.5 to 1 minimum wage), consistently with Carvalho [16], who associated non-adherence to antiretroviral treatment to the income of the surveyed families. Another study evaluated the level of adherence to treatment with antimicrobials and found that subjects with monthly family income above six minimum wages showed 8.3 times higher adherence than those with an income of five or less minimum wages[17]. Thus, income can be related to adherence mainly in extreme cases of poverty, since this condition leads to difficulty in adhering to treatment [18].

In this study, a larger family was associated with lower adherence to treatment. The number of people in the family is used in some researches to estimate family crowding, which also considers the number of rooms available in the residence. In these researches, family crowding was considered as a dynamic factor that impacts the individuals’ health [19, 20], including the adolescents’ oral health condition, as it represents a proxy variable for the social context in which they live [21]. However, there are no studies relating the number of people in the family or family crowding with adherence to dental treatment.

With this variety of factors, the health team must know the determinants that may interfere with adherence; it is imperative to recognize the specifics of their particular population. Understanding the sociocultural factors that impact adherence may help in defining what to recommend to patients, improve the communication between patient and professional, and increase adherence to the proposed treatment [22].

Health services that wish to intervene in oral health conditions caused by social inequalities must understand the inclusion oral health framework. This model describes how social exclusion, intersectionality and othering and examining care systems drivers can lead to extreme situations of vulnerability in society, and their effects on oral health.

In this sense, social assistance is essential to guarantee decent living conditions [23]. Social care can strengthen the intersectorality of actions, approaching families and their adolescents more effectively. This sector can include families in income transfer programs, helping in family planning, reinforcing the importance of children and adolescents attending schools, including guardians in programs that promote access to work, thus contributing to positively impact the social determinants that affect access to health. Specifically, social care can develop strategies that minimize the effects of vulnerabilities, which must be known and practiced by health teams.

It is important to note that the data was analyzed using multilevel analysis model, whose relevance has been pointed out by many researchers. This type of model is known by providing a more accurate assessment of the relationship between the environment and people. Probably, to date, this study is one of the first to use this technique to study factors that influence adherence to dental treatment of underprivileged adolescents.

## Limitation

The main limitation is related to the non-response rate, since we had difficulty locating important part of the sample of adolescents, although they have been sought in schools where they studied, in the Family Health Units, and also in their homes.

## Data Availability

The datasets used and/or analyzed during the current study are available from the corresponding author on reasonable request.

## Abbreviations

WHO: World Health Organization
FHU: Family Health Unit
IPPLAP: Institute for Research and Planning of Piracicaba
DMFT: Decayed, missing and filled teeth
OIDP: Oral impact on daily performance
FACES III: Family Adaptability and Cohesion Evaluation Scale

## References

[1] WHO. The World Oral Health Report (2003). Continuous improvement of oral health in the 21st century—the approach of the WHO Global Oral Health Programme.

[2] Levin L, Margvelashvili V, Bilder L, Kalandadze M, Tsintsadze N, Machtei EE (2013). Periodontal status among adolescents in Georgia. A pathfinder study Peer J.

[3] Brasil. Ministério da Saúde. Saúde Bucal Brasil 2010 (SB Brasil 2010): Levantamento Epidemiológico de Saúde Bucal: principais resultados. 2012. www.sbbrasil2010.org. Accessed 17 Mar 18.

[4] Rapoff M (2010). Adherence to pediatric medical regimens.

[5] Miller NH, Hill M, Kottke T, Ockene IS (1997). The multilevel compliance challenge: recommendations for a call to action. A statement for health care professionales. Circulation..

[6] Freddo SL, Cunha IP, Bulgareli JV, Cavalcanti YW, Pereira AC (2018). Relations of drug use and socioeconomic factors with adherence to dental treatment among adolescents. BMC Oral Health..

[7] Organização Mundial de Saúde. Cuidados inovadores para condições crônicas: componentes estruturais de ação: relatório mundial. Brasília (DF). 2003. http://www.who.int/diabetesactiononline/about/icccportuguese.pdf. Accessed 15 Oct 18.

[8] Piracicaba. Prefeitura Municipal. Secretaria Municipal de Desenvolvimento Social. Instituto de Pesquisas e Planejamento de Piracicaba (IPPLAP). http://ipplap.com.br/site/. Accessed 01 Oct 2018.

[9] Organização Mundial da Saúde. Levantamentos básicos em saúde bucal. Translation Ana Júlia Perrotti Garcia. 4.ed. São Paulo: Santos, 1999.66p.

[10] Goes PSA. The prevalence and impact of dental pain in Brazilian schoolchildren and their families. (Doctoral Dissertation). London: Department of Epidemiology and Public Health, University College London, 2001.

[11] Adulyanon S, Vourapukjaru J, Sheiham A (1996). Oral impacts affect daily performance un a low dental disease Thai population. Community Oral Epidemiol.

[12] Falceto OG, Busnell ED, Bozzetti MC (2000). Validation of diagnostic scales of family functioning for use in primary health care services. Pan Am J Public Health.

[13] Sabbah W, Tsakos G, Chandola T, Sheiham A, Watt RG (2007). Social gradients in oral and general health. J Dent Res.

[14] WHO. Social determinants of health: the solid facts. Second Edition. 2003. https://www.euro.who.int/__data/assets/pdf_file/0005/98438/e81384.pdf. Accessed 02 Aug 2020.

[15] Pessalacia JDR, Menezes ES, Massuia D (2010). The vulnerability of adolescents in a perspective of public health policies. Revista Centro Universitário São Camilo.

[16] Carvalho CV, Merchan-Hamann E, Matsushita R (2007). Determinants of antiretroviral treatment adherence in Brasília, Federal District: a case-control study. Rev Soc Bras Med Trop Uberaba.

[17] Baisch ALM, Soares MCF, Lunkes R, Goulart IDC, Silva MGC (2009). Evaluation of the treatment adherence level with anti microbial: Assistance to an educative action proposal in health. VITTALLE.

[18] Chesney MA, Morin M, Sherr L (2000). Adherence to HIV combination therapy. Soc Sci Med.

[19] Menezes PR, Scazufca M, Rodrigues LC (2000). Household and compliance with outpatient treatment in patients with non-affective functional psychoses in São Paulo, Brazil. Mann AH Soc Psychiatry Psychiatr Epidemiol.

[20] Bowie C, Pearson AL, Campbell M, Barnett R (2014). Household crowding associated with childhood otitis media hospitalisations in New Zealand. Aust N Z J Public Health.

[21] Koga R, Herkrath APCQ, Vettore MV (2020). The role of socioeconomic status and psychosocial factors on gingivitis in socially disadvantaged adolescents. J Periodontol.

[22] Kurita GP, Pimenta CAM (2004). Compliance with the treatment of chronic pain and health control locus. Rev Esc Enferm USP.

[23] Freeman R, Doughty J, Macdonald ME, Muirhead V (2020). Inclusion oral health: Advancing a theoretical framework for policy, research and practice. Community Dent Oral Epidemiol.

